# Exploring realism in high-fidelity ambulance simulation: ambulance clinicians’ perspectives on correspondence with everyday practice

**DOI:** 10.1186/s41077-026-00461-8

**Published:** 2026-07-03

**Authors:** Wivica Kauppi, Magnus Andersson Hagiwara, Hanna Maurin Söderholm, Anders Sterner

**Affiliations:** 1 PreHospen- Centre for Prehospital Research, Faculty of Caring Science, Work Life and Social Welfare, University of Borås, Borås, Sweden; 2 Faculty of Caring Science, Work Life and Social Welfare, University of Borås, Borås, Sweden; 3 PICTA- Prehospital Innovation Arena, Lindholmen Science Park, Gothenburg, Sweden

**Keywords:** High-fidelity simulation, Research, Ambulance services, Pre-hospital

## Abstract

**Background:**

High-fidelity simulation (HFS) provides a controlled environment for training, allowing ambulance clinicians’(ACs) to practice and refine their skills without risking patient safety. Additionally, it enables the replication of complex scenarios, ensuring comprehensive preparedness for real-life emergencies. However, there remains a need to understand how frontline professionals in ambulance services experience these simulation-based activities. This is especially true when using high-fidelity modalities that aim to replicate real-world scenarios for research purposes.

**Aim:**

To explore clinically active ACs´ experiences of participating in HFS.

**Methods:**

A qualitative design involving dyadic interviews with 16 ACs participating in a simulation scenario was conducted. The data were analyzed using the inductive content analysis method outlined by Elo and Kyngäs.

**Results:**

Participants emphasized the importance of adopting the right mindset from the start of the scenario, as mental readiness was crucial for immersion. Although the scenario felt realistic, achieving full psychological fidelity remained challenging, as participants’ awareness of the simulation setup influenced their behavior during the scenario.

**Conclusions:**

The study underscores the importance of carefully designed HFS scenarios that prioritize psychological and environmental fidelity to support authentic engagement among clinically active ACs. This is particularly important in pre-hospital care, where direct access to real clinical situations is often restricted by ethical, practical, and organizational constraints. The findings indicate that well constructed simulations with coherent workflow sequences, professional actor–based patient representation, and realistic settings can function as a methodological bridge for capturing complex cognitive and emotional processes that are otherwise difficult to study in real world practice. Further refinement of simulation design, focusing on psychological presence rather than complete physical or technical realism, may strengthen simulation-based research as a method for investigating experiential aspects of pre-hospital care.

**Supplementary Information:**

The online version contains supplementary material available at 10.1186/s41077-026-00461-8.

## Background

Caring for patients with breathlessness in the pre-hospital ambulance service is a scenario involving complex and rapidly changing clinical situations. Ambulance clinicians (ACs) must continuously assess, prioritize, and initiate appropriate care while attending to both physiological and emotional aspects. These demands place high requirements on clinical judgment, decision-making, and teamwork in high-pressure, unpredictable environments [1]. The scenario is one example of a complex and demanding situation that, despite the critical need for more research, is close to impossible to study in real life. Studying pre-hospital care scenarios in real-world practice is challenging due to multiple practical and ethical constraints, including limited access to the care environment, the need to safeguard patient privacy, and the difficulty of obtaining informed consent during acute, time-sensitive interventions [2–7]. High patient acuity and the need for immediate medical intervention often take precedence over research activities, limiting opportunities for data collection [8]. The inherent variability of ambulance service environments, combined with limited on-site resources and infrastructure, hinders comprehensive studies of real-world pre-hospital care processes [3, 9]. In addition, regulatory requirements and ethical approval processes vary between countries, regions, and institutions, adding further complexity for accessing the research field [10, 11]. Previous findings from our research project exploring clinically active ACs’ experiences of caring for patients with breathlessness identified key challenges in pre-hospital care [1], including clinical reasoning under time pressure, managing and communicating with patients’ relatives, and providing care in unpredictable and rapidly changing environments. These findings highlight the multifaceted nature of pre-hospital decision-making, where clinical, communicative, and contextual demands intersect. However, these complex care situations remain difficult to study in real-life ambulance settings due to ethical, practical, and organisational constraints. High-fidelity simulation (HFS) provides a method to investigate complex clinical scenarios in controlled and standardized conditions without compromising patient safety [3, 12, 13]. Fidelity refers to the extent to which a simulation replicates real-world practice and supports realism. Realism is defined as the participants’ subjective experience of the simulation as credible and representative of actual clinical work [14]. Perceived realism is crucial for participants’ immersion, yet achieving this requires thoughtful integration of technology, scenario design, and human resources [15]. Successful HFS therefore involves balancing different dimensions of fidelity, while acknowledging that not every aspect of real-world practice can be fully reproduced, but that meaningful and immersive experiences can still be created [16, 17]. Fidelity includes several elements. Physical fidelity relates to equipment, mannequins, and the physical environment. Psychological fidelity concerns participant engagement and perception of authenticity. Environmental fidelity encompasses organizational context, workflow, and available resources, while social fidelity concerns team interactions and communication [14, 18]. These elements can be enhanced through careful scenario design, appropriate use of technology, and human resources such as professional actors, all of which support realism by enabling credible patient interaction and communication [14].

HFS has been widely used in healthcare education to develop technical and non-technical skills among students [19, 20] and professionals [15]. It has also served as a tool for preparing implementation of new healthcare processes and equipment, identifying potential safety threats, and supporting the development of clinical skills and guidelines [21–25]. Within pre-hospital ambulance services contexts, HFS enables researchers to create controlled and standardized scenarios that reflect real-world pre-hospital situations without compromising patient safety or disrupting emergency care. It allows for an in-depth understanding of emergency scenarios that are difficult to study in real-world practice [3], and to test, evaluate, and improve methods, equipment, and workflows without putting patients or ACs at risk [26–28]. Despite the widespread use of HFS in training, research examining how experiences of fidelity and realism are shaped within ambulance care simulations remains limited. Most studies focus on students, educational outcomes, or skill acquisition, leaving a gap in understanding how HFS are experienced in professional ambulance care and how well they approximate real-world practice. This gap highlights the need to explore experiences of HFS that reflect pre-hospital care within ambulance services. Understanding how fidelity supports realism and correspondence with actual ambulance care practice can inform the methodological development of simulation-based research and help ensure that scenarios are authentic, clinically relevant, and suitable for empirical study. Thus, the aim of this study was to explore clinically active ACs´ experiences of participating in a HFS scenario designed to reflect pre-hospital care of patients, with a particular focus on experienced realism and correspondence with real-world ambulance care practice. The scenario was based on a patient presenting with breathlessness, as this represents a common and clinically relevant condition in ambulance care.

## Methods

### Research design

A qualitative study design with an inductive approach was applied, informed by the principles of inductive qualitative content analysis as described by Elo and Kyngäs [29]. The design was chosen to capture participants’ experiences as expressed in their own words, with an emphasis on staying close to the data and allowing meanings to emerge without predefined theoretical assumptions [29, 30]. From this perspective, the approach facilitates an understanding of ACs experiences of participating in HFS. Interviews were conducted with eight ambulance crews (*n =* 16 ACs) over two consecutive days in May and June 2021. This study constitutes one sub-study within a larger research project. While the overarching project addresses clinically active ACs´ experiences of caring for patients with breathlessness [1], the present study specifically focused on ACs experiences of participating in a HFS designed to resemble a real pre-hospital care situation involving a patient suffering from breathlessness. This study is reported in line with Cheng et al.’s (2016) guidelines for healthcare simulation research [31] (Supplementary File 1).

### Preparations

The study was inspired by the study design of Maurin Söderholm et al. (2019), which involved conducting high-fidelity pre-hospital simulations [3]. The study design was carefully planned with attention to facilities, simulation activities, and scenario development. Researchers, facilitators, and actors were selected based on their high competence and relevant expertise. The actors portrayed the roles of a simulated patient (SP) and a simulated bystander (SB). Most of the research team had extensive clinical experience in ambulance services and were well-versed in conducting HFS´s in both educational and research contexts. As they were already familiar with one another, with clearly defined roles, the implementation process was well-coordinated and effective, with reflective discussions throughout the preparation- and data collection phases.

To ensure high physical realism, insights from previous studies [32–34] informed the scenario design. The SP and SB studied the pre-written scenario and received individual instructions. The SP prepared by watching a film on breathlessness and practicing realistic symptoms. Makeup and dressing were done near the simulation site to simulate labored breathing, a pale complexion, and cyanotic lips. Two test simulations were conducted to verify the environment, equipment functionality, and scenario flow.

### Simulation width and depth

To ensure simulation *width* [3], all phases of a typical ambulance mission were included, from dispatch to the handover report via phone to an emergency department nurse; an approach previously highlighted as important in simulation design [35]. To achieve *depth*, each phase was designed for high realism [3]. The simulation design supported live role-play between participants, SP and SB, using a fully equipped ambulance with standard equipment, documentation tools, and communication devices. Sessions were conducted in a public university area during daytime, with natural background noise and movement contributing to realism [36]. While these elements could not be fully controlled, unnecessary bystander interaction was minimized. Prior to the simulation, participants could familiarize themselves with the ambulance and emergency bag but received no information about the scenario. They were instructed to act according to their usual routines, in contrast to traditional simulations that involve guided facilitation and prompts. This approach helped maintain authentic behavior and responses [37]. Each scenario began with an emergency call through a simulated dispatch system, providing initial information via radio, mimicking real-world procedures. Upon arrival, participants received typical pre-arrival details (e.g., location, symptoms). After a short transfer to the scenario site, they handled the situation as they would in real world. As recommended [38], the simulation had no fixed time limit; duration was determined by the participants. All scenarios ended with a telephone handover report to a fictitious emergency department.

### Research- and simulation equipment

During the scenarios, live-stream video was used for remote observation to prevent researchers from disturbing participating crews. Cameras were positioned in three locations inside the ambulance. A simulation facilitator stationed nearby provided real-time vital parameters (VPs) (oxygen saturation, blood pressure, respiratory rate, and heart rate) via an iPad connected to a portable monitoring device, which also displayed electrocardiogram (ECG) waveforms. VPs, ECG, and blood test results were shown on the monitor only when participants initiated monitoring or performed fictitious blood tests. The facilitator stayed nearby to support the crew when needed, while maintaining a respectful distance to avoid interference. A stethoscope with pre-programmed obstructive lung sounds was also available to enhance realism.

### Participants

Participants were recruited from two ambulance organizations in southwestern Sweden, covering urban to rural areas. In Sweden, ambulance crews include at least one registered nurse (RN), usually with specialist training in pre-hospital, intensive, or anesthesia care, and often an ambulance technician with assistant nurse training. The term *ambulance clinicians* (ACs) refers to all care providers, regardless of professional background. Participants were recruited through purposive and snowball sampling. Inclusion criteria required RNs with specialist training in ambulance-, intensive-or anesthesia care. Efforts were made to ensure variation in age, gender, work experience, and geographic location. After receiving approval from chief executive officers, recruitment was carried out via internal digital platforms, social media forums, and personal invitations from participants and research team. Interested individuals contacted the first author to receive more information and confirm eligibility. Sixteen participants (11 men, 5 women) participated in the study, representing a variety of ambulance stations and care contexts, contributing to a rich and diverse understanding of ambulance services (see Table 1 for participant characteristics). No participants withdrew from the study.

**Table 1:** Characteristics of included participants

	*n* = 16
Gender	
Male	11
Female	5
Age (years)	
25–34	5
35–44	5
45–54	6
Ambulance service clinical experience (years)	
0–3 years	3
4–6 years	6
7–10 years	2
11–15 years	2
16–22 years	3
Specialization areas of registered nurses	
Ambulance care	12
Intensive care	1
Anesthesia care	1
Ambulance- and Intensive care (dual competence)	1
Anesthesia- and Intensive care (dual competence)	1
Experience in workplace-based simulation (excluding skills training)	
Yes	13
No	3
Experience in educational program simulation (excluding skills training)	
Yes	7
No	9

### Data collection

Data were collected through eight dyadic [39], semi-structured interviews, in which the same pairs of participants who completed the simulation were interviewed together immediately afterward in a nearby facility. Each interview lasted 10–20 min (median 16) (Questions are presented in Table 2). The interviews were conducted by WK (*n* = 5), HMS (*n* = 1), and an experienced external researcher (*n* = 2). All interviews were audio-recorded and later transcribed verbatim by an external transcription service.

**Table 2:** Overview of interview questions used in the study

Interview question
• What did you find to be the most effective aspect of the simulation’s structure?
• Was the equipment in the ambulance and the bag with accessories and medications similar to what you are used to?
• Before you conducted the scenario, did you have sufficient time to familiarize yourself with the bag and equipment?
• Was there anything that was difficult to understand how to use during the simulation?
• Did you encounter any situations during the simulation where you were unsure of how to proceed in the scenario?
• Could you work as you normally do, even during the simulation?
• Was this a typical patient case that you have encountered before?
• Is there anything we can improve or do differently regarding the simulation itself?
Examples of follow up questions to deepen responses
• Can you describe that in more detail?
• Can you give a concrete example?
• How did it affect you?
• What do you think contributed to that?

### Data analysis

**Fig. 1:**
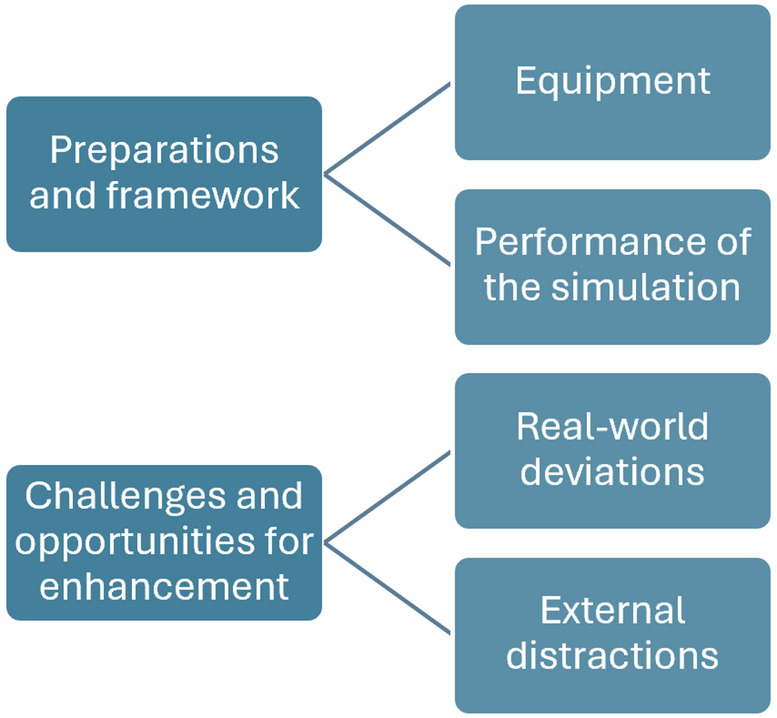
Description of generic categories and subcategories

The data were analyzed using Elo and Kyngäs (2008) approach to inductive content analysis. A manifest, text-near approach was applied throughout the analysis. The process followed these steps: (1) making sense of the data through reading the transcripts numerous times, (2) open coding to describe all aspects of the content, (3) grouping codes to subcategories, (4) grouping subcategories under higher order headings into broader generic categories. This process was iterative and discussed between the first and last author. When the results were presented to the research group, categories were discussed again and consensus was reached. In all, the analysis revealed four subcategories and two generic categories (see Fig. 1).

### Rigor

To ensure credibility and trustworthiness [30], all authors (WK, MAH, HMS and AS) engaged in continuous reflexive discussions throughout the study to identify and critically examine their pre-understandings and potential biases. These reflections were addressed by explicitly questioning emerging interpretations, comparing coding decisions among authors, and repeatedly revisiting the data to ensure that the findings were grounded in participants’ accounts rather than researchers’ assumptions. Key aspects of this reflexive process have been made visible to the reader in both the Methods and Discussion sections. The analysis was conducted manually in accordance with the principles of an inductive approach [29]. Initial coding and categorization were performed by AS, while WK critically reviewed the coding framework and the emerging categories. Discrepancies were discussed among AS and WK until consensus was reached, strengthening dependability and transparency. Although AS did not participate in data collection, this author contributed extensive experience in qualitative research and simulation in both educational and research contexts, thereby supporting analytical rigor. All steps of the analysis were systematically documented, and representative interview quotes were included to support the results and facilitate transferability.

### Ethical considerations

This study was approved by the Swedish Ethical Review Authority (Dnr 2019–03283) and conducted in line with the Helsinki Declaration [40]. All participants received written and verbal information about the study and gave informed consent. Participation was voluntary, with the right to withdraw at any time. After transcription, the audio recordings were securely stored with access limited to the study authors. All data collection and storage complied with the General Data Protection Regulation (GDPR) and the university’s internal guidelines.

## Results

The results are based on 124 min of audio-recorded interview data generated from eight dyadic interviews with clinically active ACs´. The results are presented in four subcategories and two generic categories that illustrate ACs experiences of participating in a HFS scenario. Figure 1 provides an overview of these categories, while the results section elaborates on each subcategory using illustrative quotations from ACs.

### Preparations and framework

The simulation’s preparation and framework were experienced by ACs as clear and straightforward, ensuring that they understood what was expected of them. A prebriefing session outlined its purpose, expectations, and procedures. The opportunity to perform tasks authentically, as they would in real-world practice was appreciated, enhancing immersion and the sense of working in a real-world clinical environment.

### Equipment

After the prebriefing, ACs familiarized themselves with the equipment and the ambulance. They noted that two types of equipment deviated significantly from standard practice: one was the stethoscope that could play respiratory sounds during auscultation, and the other was the monitoring device. Familiarizing with the equipment prior to the simulation was considered crucial by the ACs in reducing stress during the simulation. The equipment was familiar to the ACs, although some differences were noted. At the same time, ACs likened using the new emergency bag to changing workplaces, others required additional time to locate the equipment. This did not hinder their ability to work effectively as they would in a clinical setting, nor did it affect their overall simulation experience.

*“With my usual bag*,* I can grab the right items with my eyes closed. Here*,* I might have to search a bit more*,* even if I look at it beforehand. But honestly*,* it doesn’t affect the simulation that much. At least*,* that’s what I think”* (Alex, Interview 1).

### Performance of the simulation

ACs emphasized that the SP and SB, portrayed by actors, contributed to enhancing the simulation experience. Even ACs who sometimes found it difficult to engage in simulations experienced that having a live, trained actor portraying the SP significantly enhanced immersion. When ACs entered and saw the surroundings and the SP, an immediate condition for performing an initial patient assessment was established, even from a distance.

*“It was good in every way*,* she [the SP] was in a tripod position and used respiratory muscles and stuff. So you understand that ‘okay*,* she’s having difficulty breathing’.. you could see that from a distance”* (Robert, Interview 5).

According to some ACs, the use of an SP and SB in the simulation resulted in a distinctly different experience compared to scenarios involving mannequins. This was an element the ACs had previously identified as lacking in mannequin-based simulations. The fact that the SP was entirely unfamiliar to them was perceived as a positive aspect. There were no recognizable cues that could interfere with the immersive experience of managing a patient in the presence of a distressed family member. Moreover, ACs reported that the presence of a live actor portraying the SP facilitated both communication and the conduct of the patient interview, as responses and feedback were provided in real time. It became easier to acknowledge and respond to the SP individual needs in care. Aspects such as the need for privacy became more evident to ACs when the immersive nature of providing care in a realistic clinical setting was made clear.

*“When you’re in a workplace*,* you want some level of privacy*,* and that can be harder to experience in a school setting when there are people standing around watching. So*,* I think it was actually good that we knew we were going to a workplace and that it was actually taking place in a real workplace” (*Robert, Interview 5).

For most ACs, this type of simulation was a new experience, but they viewed the realistic design positively. Using an actor and the fully equipped ambulance made the scenario feel refined and credible, helping them think and act as they would in real patient care. It also allowed them to make real-world decisions, such as providing care on-site or in the ambulance.

*”It felt like a regular patient case..that’s really how it felt”* (Daniel, Interview 1).

### Challenges and opportunities for enhancement

Although the simulation framework was clear and well-received, some ACs identified potential disruptions and areas for improvement. To further enhance immersion, the incorporation of elements such as ambulance driving, along with other aspects differing from participants’ routine clinical practice, was suggested to create a more authentic simulation experience.

### Real-world deviations

One aspect highlighted by the ACs was the need to create a time buffer between the prebriefing and the start of the scenario. In a real-world dispatch call, a chain of tasks and events begins that affects the ambulance crew during the period between the dispatch and their arrival at the patient. Some ACs expressed a desire for the dispatch to function as it does in daily practice, in order to enhance immersion and help them get into the same mindset as during a real ambulance assignment. This would involve the alarm requiring active acknowledgment by the dispatch center. ACs experienced that relevant information would be displayed on a screen in the ambulance and could be continuously updated during transit as new data is received. This setup would ensure that the crew receives real-world updates en route to the scene.

*“..then it gets a bit easier to get into the work*,* kind of”* (Alex, Interview 1).

Several ACs highlighted the absence of ambulance driving during the simulation as a significant limitation. One suggestion was to begin the scenario a few hundred meters from the simulation site, or to have the crew drive and reverse into the designated area. This omission excluded a key component of the alarm chain and affected the interaction and communication between ACs during transport. These roles carry distinct demands, which may be overlooked when the related tasks are not included in the simulation. This was particularly relevant for the driver, who typically has fewer opportunities to engage in preparatory discussions compared to the caregiver. Moreover, ACs emphasized that in real-world situations driving an ambulance can be a considerable source of stress, whether due to speed, traffic conditions, or difficulties in locating the correct address. This often influences the team dynamic, resulting in reduced communication and a tendency for planning to occur ad hoc upon arrival.

*“ Then you grab the bags and sort of make the plan during those 40 steps in…”* (Adam, Interview 3).

Upon arriving at the SP, ACs experienced that the usual stress response present in real-world situations was missing, resulting in a reduced sense of urgency. ACs described how ambulance personnel often encounter a sense of panic from relatives, conveyed through body language or facial expressions, when arriving at a scene. During the simulation, this was primarily expressed through words, which did not fully recreate the atmosphere of real-world situations. At the same time, ACs acknowledged that replicating such emotions in a simulated environment can be challenging. Additionally, the simulation presented challenges, particularly in the initial assessment of the SP who suffered from breathlessness. ACs noted that, based on previous work experience, such patients are often more severely affected than the SP could portray. While breath sounds were audible through the stethoscope, real-world cases often allow these sounds to be heard from a distance as they approach the patient.

*“But you don’t hear the wheezing*,* you don’t hear the hoarseness*,* you don’t really hear the fast breathing. She’s not actually sick”* (Robert, Interview 5).

A technical challenge according to ACs was the variation in documentation methods across participating ambulance stations. While several ACs had prior experience of various systems, most ACs concluded that this did not significantly impact their performance during the simulation. Additionally, a highlighted technical difference was the transfer of patient data to the monitor. For example, temperature readings were provided by a facilitator, requiring ACs to verbalize to a non-existent person to receive the information. This method of conveying patient data also limited their ability to assess respiratory rate, take manual blood pressure, or feel and evaluate the pulse during the assessment. Among ACs with extended work experience, certain aspects of patient assessment were so automated and deeply embedded in their routines that the limitations of the simulation became evident.

*“..so when I approach the patient and start talking*,* I’m holding her wrist and listening to how she breathes… and I do that during the first thirty seconds of the interview*,* kind of. And that’s not something we normally.. talk about out loud”* (Adam, Interview 3).

Moreover, ACs emphasized that there was a risk that the SP’s visual presentation did not fully correspond with the provided vital parameters.

*“Even though she acted really well*,* you kind of forget that you’re supposed to look at this screen…and then it becomes.. well*, *it feels a bit unnatural”* (Laura, Interview 7).

The most significant difference in equipment noted by ACs was related to the medications used in the simulation. Since the medication vials were not real, their size, appearance, and labeling differed from real-world. Another aspect was that certain medical procedures could not be performed, which may have impacted immersion according to ACs. For example, they noted that the oxygen tank contained no gas, peripheral intravenous cannulas were not inserted, and medications were not administered in the way they would be in a real-world practice.

*“I guess I have a bit more difficulty immersing myself in the situation*,* in a way. When you’re not actually… well*,* you’re not putting in the needle or connecting the electrocardiogram.. it’s obviously a bit different for me*,* somehow”* (Robert, Interview 5).

ACs previous experiences with simulation emerged as a potential obstacle to working in the same way they would in clinical practice. This was particularly evident among ACs with prior experience in simulations during previous educational programs, where scenarios often became increasingly complex to challenge them as part of their student roles. This created an expectation among ACs that the simulation would also evolve in a similar way. They anticipated an upcoming “twist” in the scenario and adjusted their approach accordingly, maintaining a certain level of preparedness for unexpected developments.

*“There’s supposed to be a progression all the time. It should always start a bit easier*,* and then as you go on… but there wasn’t really any such progression”* (Daniel, Interview 1).

### External distractions

 The fact that some ACs felt they were being observed, despite this not being the intention of the simulation, could not be entirely disregarded. Some ACs felt they might have spoken more and worked at a slower pace than they would have in a real-world practice.

*“I mean.. I think you check things off faster in a simulation than you might in real life.. you know”* (Robert, Interview 5).

The presence of public people standing around or moving close by the simulation site was not perceived as a problem among ACs, as it resembled real-world practice. They also felt that since the cameras were small and permanently mounted in the ambulance, they did not affect the environment in the same way as if someone had been filming directly. However, ACs were aware of the cameras, and knowing they were being filmed sometimes led them to adjust their movement, adapting to the camera’s position or their equipment.

*“Yeah*,* but you kind of do that all the time [feeling watched by the camera].. so I think it’s hard to shake off the feeling of being watched”* (Michael, Interview 7).

## Discussion

This study explores HFS as a pre-hospital research method and environment, based on clinically active ACs’ experiences of participating in an HFS scenario. Participants’ accounts illuminate how different fidelity dimensions interact in pre-hospital HFS and suggest that, for clinically active ACs, psychological and environmental fidelity often outweigh perfect physical fidelity. By focusing on participants who are clinically active rather than students, this study addresses a gap in the literature and demonstrates that HFS can effectively elicit experiential knowledge in pre-hospital care. Participants provided insights into how realism is experienced in a research setting and how it influences their engagement and clinical reasoning.

### Fidelity in professional research contexts

Participants’ reflections revealed how different dimensions of simulation fidelity interact in pre-hospital HFS designed for research purposes. In the category “Preparations and framework,” participants highlighted physical fidelity, including equipment and a fully equipped ambulance vehicle, environmental fidelity, such as realistic outdoor settings with natural background noise, and a complete workflow from dispatch to handover, as well as social fidelity, supported by professional actors enabling authentic patient interaction and realistic team dynamics. In the category “Challenges and opportunities for enhancement,” participants described tensions between these dimensions and psychological fidelity. They emphasized that the scenario’s credibility depended on its ability to evoke authentic interaction by the SP, clinical reasoning, and emotional responses. Lavoie et al.’s framework of practical fidelity features [14] helps situate the findings within the broader simulation literature. Human actors are critical for realism, equipment familiarization corresponds to the “preparation of environment” feature, and the complete workflow reflects the “logical and adaptive scenarios” dimension. However, our findings extend this by highlighting that clinically active ACs have distinct fidelity requirements compared to students, e.g. the need to verbalize tacit physical assessments (e.g., “I am checking the pulse”) introducing artificial cognitive demands not present in real care. This suggests that fidelity frameworks developed for education may need adaptation for research, where simulation is used as a research environment and the goal is to capture authentic decision-making rather than assess explicit knowledge [14].

### Width vs. depth, mental readiness and disruptions

Mental readiness, a core aspect of psychological fidelity, is shaped by factors such as how patients are represented and the authenticity of the simulation environment. Previous studies suggest that using actors instead of manikins enhances realism [14, 41, 42]. In this study an actor portrayed the SP, which contributed to immersion, but the lack of real-time VP such as those seen in actual cases of breathlessness limited the sense of realism. While previous studies highlight the challenges of replicating high-fidelity pre-hospital simulations due to cost and technical complexity [43], the integration of wearable technology that displays dynamic VP on monitors may offer a feasible solution to enhance contextual authenticity while acknowledging cost and technical challenges.

Participants in our study emphasized that entering the right mindset from the start of the scenario was critical. They described how, in real practice, dispatch and driving create a preparatory phase for mental readiness, role clarification, and initial reasoning. Participants reflected that starting close to the scene removed that transition, which may have limited psychological fidelity. These observations are consistent with previous results showing that environmental consistency and coherent temporal progression are central to immersion, while abrupt shifts can disrupt engagement [36, 44–46], for participants to remain immersed in a simulation, events must follow a logical and realistic flow. A potential improvement for future studies is to start the simulation from a separate location, allowing ACs to respond and drive to the scene, better mirroring real-world workflow and enhancing situational buildup. Another option could be a purpose-built ambulance that enables simulated driving to the patient.

### Fiction contract and hierarchy of fidelity

In our study, participants emphasized the importance of a fiction contract, in line with the descriptions by Dieckmann et al. [47] and Rudolph et al. [48]. They engaged “as if” the scenario were real despite obvious unrealistic elements, supported by clear framing and a psychologically safe environment. Participants described which fidelity elements were essential for maintaining this contract and which deviations could be tolerated. They accepted surface-level physical deviations, such as simulated medication vials, unfamiliar equipment layouts likened to “changing workplaces”, and small mounted cameras, which were experienced as imperfect but not disruptive. However, participants reported that deviations disrupting temporal flow or embodied routines, such as the absence of a dispatch phase, lack of ambulance driving, insufficiently acute patient presentation, or the requirement to verbalize assessments, were more disruptive to psychological fidelity and the scenario’s correspondence with real practice. Participants’ reflections suggest a hierarchy of fidelity investments for simulation-based research. They emphasized that, when resources are limited, prioritizing simulation width, including a complete workflow from dispatch and driving to on-scene care and handover, and emphasizing social and environmental fidelity, such as authentic settings, skilled actors, and realistic team interaction, may yield greater gains in psychological fidelity than investing in perfect physical depth. An important exception, as participants noted, is technology that allows dynamic, real-time VP than requiring verbal requests to facilitators.

These findings highlight the practical trade-offs that should be considered when designing simulations for research purposes [3]. Participant’s accounts illustrate how fidelity is not solely dependent on technical features but also on how well the scenario captures the situational demands and clinical complexity of pre-hospital care. When developing future simulation scenarios for research purposes, careful consideration is therefore needed regarding how width and depth are balanced against practical constraints such as time, resources, and logistical feasibility. The resource–fidelity trade-off becomes particularly important when simulation serves as a research environment rather than an educational intervention. The goal is not skill acquisition or performance assessment but eliciting authentic experiential knowledge and enabling rich, reflective data generation.

### Implications for future research

These findings suggest that “good enough” realism for research does not require multi-million dollar facilities. Instead, coherent narrative flow and sufficient psychological and environmental fidelity can trigger genuine clinical reasoning and emotional engagement, even if some physical procedures remain simulated. Future methodological studies could systematically vary fidelity elements and explore participants’ responses. For example, researchers could test whether increasing simulation width, such as adding the drive, produces richer qualitative data than increasing depth, such as using real fluids or medications. Participants’ insights could help establish metrics and standards for pre-hospital research simulations and determine which combinations maximize perceived realism, engagement, and quality of experiential data relative to cost and logistical complexity.

### Limitations

This study has several limitations to consider. Only a single HFS scenario, focusing on the care of patients experiencing breathlessness, was used. Developing realistic simulations for such patients is resource-intensive, which can limit scenario complexity and reproducibility. Data were collected through dyadic interviews conducted immediately following the simulation. This timing may have influenced participants’ responses, as the presence of a colleague, heightened emotional states, limited time for reflection, and a tendency toward socially desirable responses could have affected what was shared. At the same time, dyadic interviews facilitated richer insights by capturing shared experiences and enabling participants to build on each other’s reflections, which may have enhanced the depth of the data [49]. Participants may also have emphasized the most striking or stressful aspects of the scenario. Furthermore, prior experience with simulation could have shaped their perceptions of fidelity and realism. The analysis mainly focused on manifest content, but some attention to latent content was unavoidable during categorization in order to gain a deeper understanding, which can be seen as a strength [50]. This approach also carries the risk that researchers’ preconceptions shaped the interpretation. To mitigate this, the authors engaged in ongoing reflexive discussions, explicitly questioning their assumptions and revisiting the interview data to ensure that interpretations were grounded in participants’ descriptions. In addition, summarizing the data into categories may have led to a loss of some subtle details in the participants’ experiences. Despite these limitations, the study provides valuable insights into how clinically active ACs´ experience HFS, which can inform future scenario development and research.

## Conclusion

This study provides insight into how clinically active ACs´ experience fidelity and realism in HFS designed for research, a perspective largely overlooked in pre-hospital simulation literature that has focused mainly on students and training. The findings highlight the value of carefully constructed simulation scenarios and the importance of psychological fidelity for enabling authentic engagement, including actor-based patient representation, complete workflow sequences, and environmental authenticity experienced as more critical for maintaining than perfect props, uniform equipment, or full procedural realism. The results extend existing fidelity frameworks suggesting that psychological and environmental fidelity can partly compensate for limitations in physical fidelity when resources are constrained, while specific gaps are experienced as particularly disruptive to immersion and correspondence with real practice.

These insights underscore simulations’ potential as a developing research method for studying experiential, emotional, and cognitive aspects, particularly in healthcare contexts with limited direct access such as ambulance work. Future research could refine simulation design and explore its ability to capture complex emotional and cognitive states. This could be achieved, for example, by varying key fidelity elements and examining their effects on psychological fidelity, participant engagement, and the richness of qualitative data.

## Supplementary Information

Supplementary Material 1.

## Data Availability

The data that support the findings of this study are not openly available due to reasons of sensitivity and are available from the corresponding author upon reasonable request. Data are located in controlled access data storage at the University of Borås, Sweden.

## Abbreviations

AC: Ambulance clinician
ECG: Electrocardiogram
HFS: High-fidelity simulation
RN: Registered nurse
SB: Simulated bystander
SP: Simulated patient
VP: Vital parameter
