# Supplementary material for: Exploring realism in high-fidelity ambulance simulation: ambulance clinicians’ perspectives on correspondence with everyday practice
Source: Adv Simul (Lond). 2026 Jul 3;11:48. doi: 10.1186/s41077-026-00461-8 (PMC13332597; doi:10.1186/s41077-026-00461-8)
Supplement: Supplementary file 1 — Supplementary Material 1. [file 41077_2026_461_MOESM1_ESM.docx]

Supplementary File 1. Checklist for reporting transparency

| **Elements** | **Subelements^1^** | **Descriptor** |
| --- | --- | --- |
| Participant orientation | Orientation to the simulator | Prior to the simulation, participants could familiarize themselves with the ambulance and emergency bag but received no information about the scenario. |
| Simulator type | Simulator make and model | Not applicable – in situ simulation using a real ambulance and human actors. |
|  | Simulator functionality | Physiological responses were portrayed by the simulated patient; no mannequin-based functionality was used. |
| Simulation environment | Location | Sessions were conducted in a public university area during daytime. |
|  | Equipment | Fully equipped ambulance with standard equipment, documentation tools, communication devices and emergency bag |
|  | External stimuli | Natural background noise and movement. |
| Simulation event/scenario | Event description | Each scenario began with an emergency call through a simulated dispatch system, providing initial information via radio. Upon arrival, participants received typical pre-arrival details (e.g., location, symptoms). After a short transfer to the scenario site, they handled the situation as they would in real life. |
|  | Group vs. individual practice | In pairs (two). |
|  | Use of adjuncts | Adjuncts included actor moulage (cyanotic lips, pallor), radio dispatch communication, and standard ambulance equipment. |
|  | Facilitator/operator characteristics | Facilitator was a researcher and expert in prehospital emergency care at the present university. |
|  | Pilot testing | Two independent tests were performed before the study session. |
|  | Actors/confederates/standardized/simulated patients | Simulated patient by professional female actor. The patient actor prepared by watching a film on breathlessness and practicing realistic symptoms. Makeup and dressing were done near the simulation site to simulate laboured breathing, a pale complexion, and cyanotic lips. |
| Instructional design (for educational interventions) or exposure (for simulation as investigative methodology) | Duration | No fixed time limit. |
|  | Frequency/repetitions | One session for each pair. |
|  | Clinical variation | One patient experiencing breathlessness. |
|  | Standards/assessment | Work according to their usual routines. |
| Feedback and/or debriefing | Source | No feedback or debriefing was provided, as the simulation was used solely as an investigative methodology. |
|  | Video | Yes, for recording purpose only. |

Note. Checklist based on Table 3 in Cheng et al. (2016) [31]. Not all items were applicable to the present study.

Description is required only if applicable^1^
